# Prevalence of malnutrition and associated factors among the elderly population in Jiaxing City, China

**DOI:** 10.3389/fnut.2025.1699703

**Published:** 2026-02-17

**Authors:** Yangming Sun, Zirui Wang, Yikang Wu, Yun Lin

**Affiliations:** 1 Department of Nutrition and Food Safety, Jiaxing Center for Disease Control and Prevention, Jiaxing, Zhejiang, China; 2 Department of Nutrition and Food Safety, Taizhou Municipal Center for Disease Control and Prevention, Taizhou, Zhejiang, China

**Keywords:** elderly care, mini nutritional assessment, influencing factors, malnutrition, regression analysis

## Abstract

**Objective:**

Analysis on the nutritional status and its influencing factors of the elderly in care institutions in Jiaxing.

**Method:**

The Mini Nutritional Assessment (MNA) was employed to screen the risk of malnutrition among individuals aged 60 years and older residing in these institutions. The single-factor and multi-factor logistic regression models were utilized to analyze the influencing factors.

**Results:**

A total of 373 subjects were included in this survey, and the age group of 80–89 years is the largest, accounting for 50.40%, while the 60–69 years age group is the smallest, accounting for 9.12%. One hundred seventeen subjects were at risk of malnutrition, accounting for 31.37% of total subjects. Multivariate regression analysis showed that the risk of malnutrition among the elderly in privately run and publicly owned care institutions is 0.19 times that of those in publicly constructed and owned care institutions (95% CI: 0.07–0.52). The risk of malnutrition among the elderly with the ability to go out independently is 0.01 times that of those bedridden individuals (95% CI: 0.00–0.07). The risk of malnutrition among the elderly without peptic ulcer is 0.39 times that in the affected individuals (95% CI: 0.16–0.98).

**Conclusion:**

The screening results for malnutrition risk among the elderly individuals in care institutions indicate that the proportion of elderly individuals at risk of malnutrition is relatively high. The risk of malnutrition is particularly increased for bedridden individuals and for those suffering from peptic ulcers.

## 1 Introduction

With improvements in medical conditions, social security, and living standards, China’s mortality rate has declined, life expectancy has increased, and the aging population problem has become increasingly serious. As of the end of 2018, the elderly population aged 60 and above in China was about 250 million, accounting for 17.9% of the total population, which indicates that the country has entered a significantly aging society (1). In the past three decades, China’s aging population has become a serious problem, with the proportion of elderly individuals increasing (2). Additionally, the health status of the elderly is concerning. Hypertension, bone and joint diseases, cardiovascular and cerebrovascular diseases, gastropathy, and diabetes have become the main chronic conditions affecting this population (3).

Nutritional status is crucial among the multiple factors that contribute to the health of the elderly. Malnutrition is often referred to as ‘hidden hunger’ and its causes include inadequate food intake, disease, and the aging process (4). As older adults may lose their ability to care for themselves, and their chewing and digestive abilities may decline, which can affect their nutritional intake and significantly increase the risk of malnutrition. Older adults are particularly susceptible to malnutrition, which raises the risk of adverse clinical outcomes such as death, functional decline, and infection (5, 6). The incidence of periodontal disease among the elderly is on the rise, the tooth loss is worsening. Problems such as gum swelling and recession can also cause difficulty in chewing. The aging process is often accompanied by a decrease in saliva flow or changes in saliva composition. Elderly individuals may also experience swallowing disorders, drooling, and reduced chewing frequency (7). Chewing and swallowing disorders can affect eating in elderly individuals, leading to problems such as insufficient nutrient intake and malnutrition. Malnutrition, in turn, affects the health and functional status, and these two factors interact and reinforce each other, gradually forming a vicious cycle. Therefore, improving nutritional status can bring tangible benefits to the elderly, as many age-related diseases and conditions can be prevented, managed, and improved through proper nutrition and dietary management (8, 9).

In recent years, a large number of welfare homes, nursing homes, senior apartments, sanatoriums, and elderly rehabilitation centers sponsored by civil affairs departments and non-governmental organizations have emerged. Many elderly individuals choose to stay in these elderly care service institutions, and there is a greater demand for disabled elderly individuals (10, 11). However, due to various factors such as uneven service quality and inadequate professional skills among nursing staff, elderly care institutions face challenges, including unbalanced dietary intake, limited physical function, and an increased risk of malnutrition. Studies have shown that elderly individuals who live at home tend to have better health conditions than those residing in elderly care institutions (12). Therefore, conducting malnutrition screening for elderly individuals in care institutions is essential. Early identification of malnutrition should be implemented to provide a basis for further nutritional interventions (13, 14).

## 2 Methods

### 2.1 Research subjects

**Figure 1:**
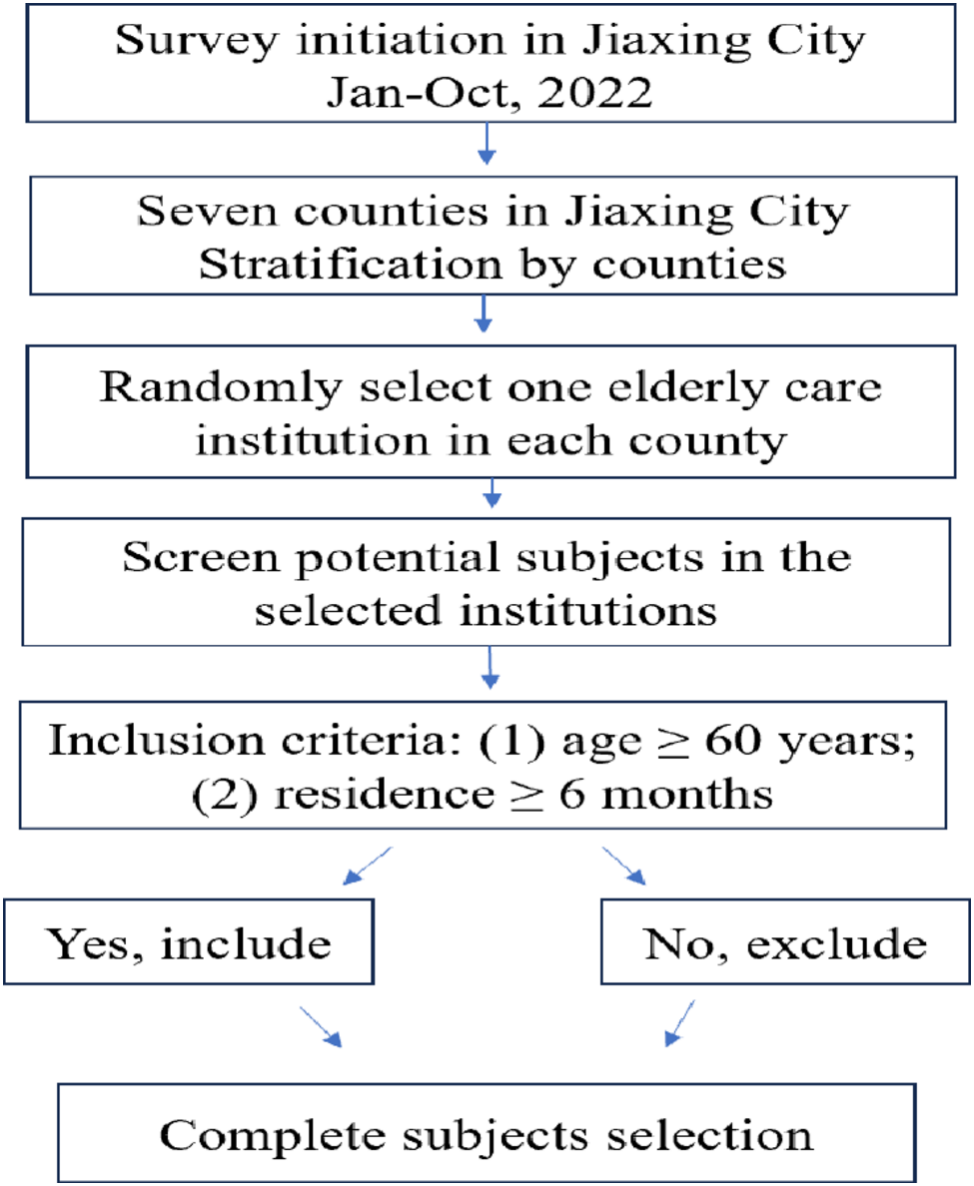
The flowchart of research subjects selection.

A stratified cluster sampling method was used, with counties as the strata. From January to October 2022, one elderly care institution was randomly selected in each of the seven counties under the jurisdiction of Jiaxing City for the survey. The inclusion criteria for the research subjects were: (1) age ≥ 60 years; (2) residence in the elderly care institution for ≥ 6 months (Figure 1). The sample size was calculated using epi info software (version 7.2.7.0), with acceptable margin error of 5%, expected frequency of 30%, and an estimated sample size of 322 individuals approximately. During the survey, 388 elderly individuals participated in the study after providing informed consent. However, 15 individuals provided incomplete survey questionnaire information. Ultimately, 373 individuals were included in the survey analysis.

### 2.2 Research methods

Before launching the on-site survey, the surveyors received unified training. With the cooperation of the elderly care institution managers, face-to-face surveys were conducted with the research subjects. The survey content included: (1) Basic information about the institution: such as the type of institution and the total number of elderly residents; (2) Nutrition risk screening, utilizing the internationally accepted Mini Nutritional Assessment (MNA) questionnaire to assess the nutritional risk of the elderly residents. The MNA questionnaire, first proposed by Guigoz for the nutritional assessment of the elderly, is a simple and non-invasive method that is linearly correlated with traditional nutrition evaluation methods. The MNA questionnaire is divided into two parts: screening and assessment. Research subjects with screening scores of ≥12 are considered to have good nutritional status and do not need further assessment. Those with scores of <12 will continue to be assessed. The sum of scores from the screening and assessment components indicates good nutritional status if it is ≥24, a risk of malnutrition if it is between 17 and 24, and definite malnutrition if it is <17. The elderly who are at risk of malnutrition, as well as those with definite malnutrition, are categorized together into the malnutrition group.

### 2.3 Quality control

The on-site survey received technical support from the Zhejiang provincial center for disease control and prevention, and all surveyors involved were professional technicians. Prior to the survey, all surveyors underwent standardized training. Data collection was conducted using paper questionnaires, and the collected data were subsequently entered into Epidata 3.0 software to establish a database. To ensure accuracy and reliability, double data entry and consistency checks were performed.

### 2.4 Statistical methods

The software of Epidata 3.0 was used for data entry, the sample size was calculated using epi info software (version 7.2.7.0), and the survey data were analyzed descriptively using R software (version 4.3.3). The univariate and multivariate logistic regression analysis were also used R software to test the statistical significance of the regression coefficients of each variable and determine whether their impact on the dependent variable is statistically significant. The nutritional status of elderly individuals was treated as a binary dependent variable, and variables that showed significant results in the univariate analysis were included in the multivariate analysis. Stepwise logistic regression was used for the multivariate analysis, with a *p*-value of <0.05 considered statistically significant.

## 3 Results

### 3.1 Basic information

**Figure 2:**
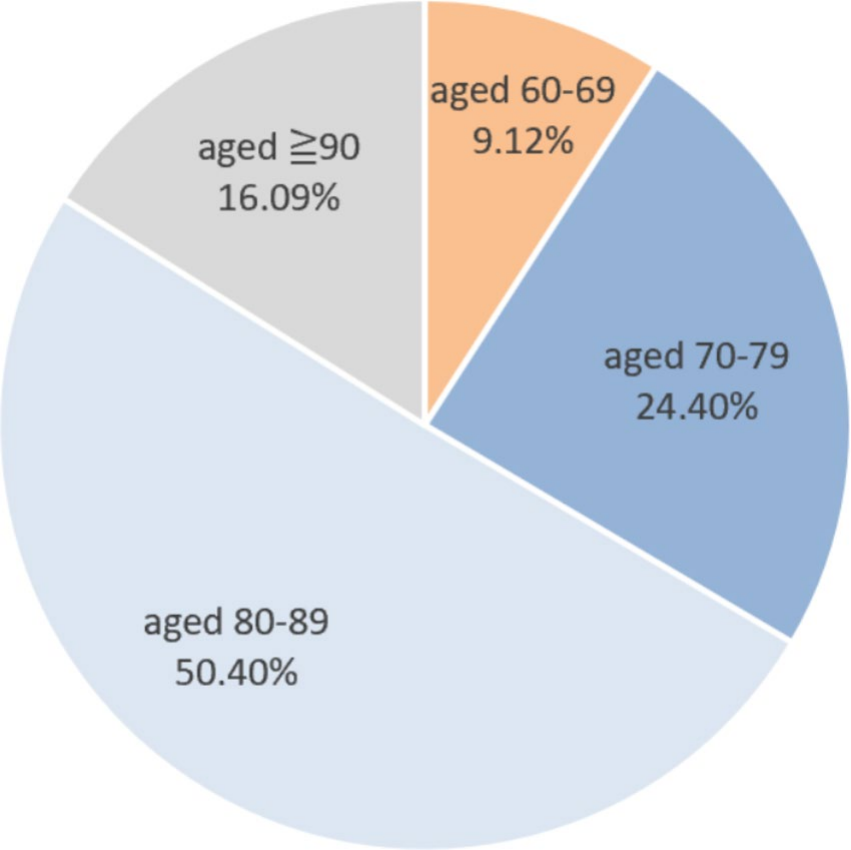
The age proportion of research subjects.

**Figure 3:**
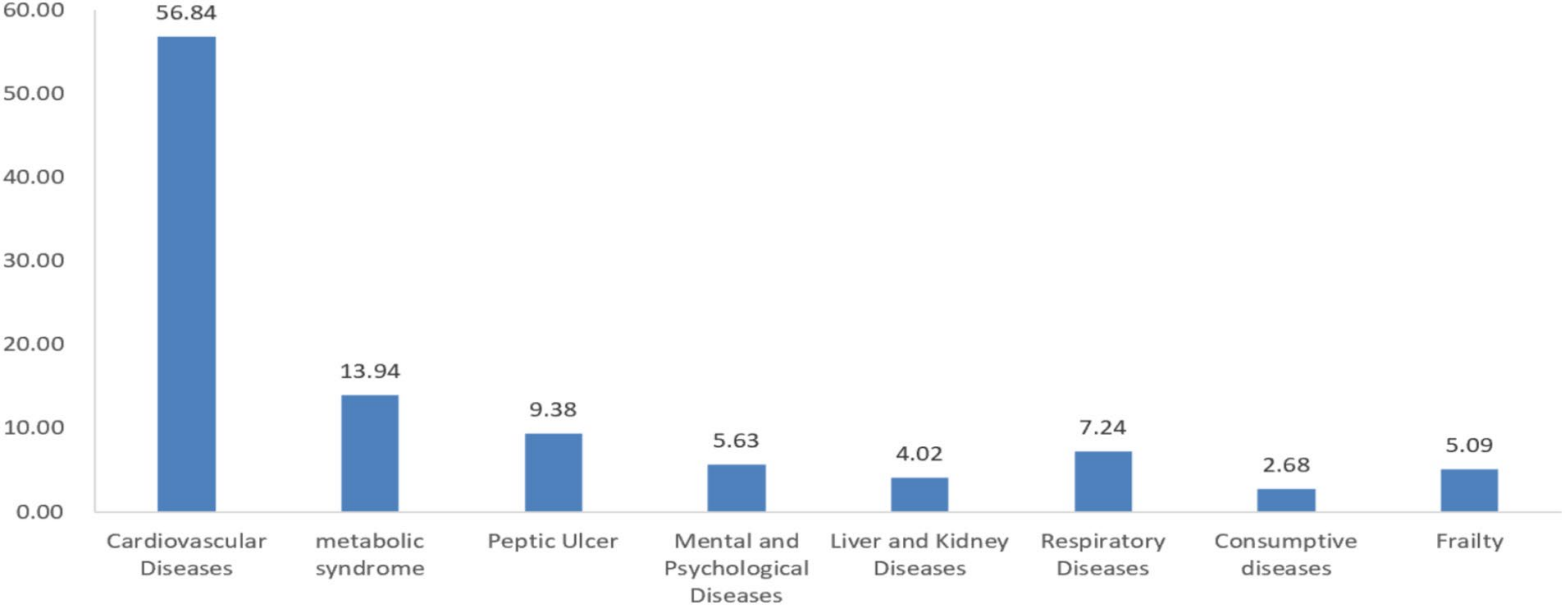
The prevalence rate of chronic disease among research subjects (%).

A total of 373 research subjects participated in the survey according to the inclusion criteria. Of these, 197 were male (52.82%) and 176 were female (47.18%). The age group with the highest proportion was 80–89 years (50.40%), followed by 70–79 years (24.40%), ≥90 years (16.09%), and 60–69 years (9.12%). In terms of the types of elderly care institutions, 154 subjects were from privately run and publicly owned institutions (41.29%), while those from publicly constructed and owned, publicly constructed and privately owned, and privately run and privately owned institutions accounted for 29.76, 14.75, and 14.21%, respectively. Additionally, 56.84% of the research subjects had cardiovascular and cerebrovascular diseases, while 13.94% had metabolic syndrome. The proportion of subjects with other adverse health conditions, such as mental and psychological disorders, liver and kidney diseases, peptic ulcers, and respiratory diseases, was less than 10% (Figures 2, 3).

### 3.2 Nutrition status

The MNA questionnaire was used to screen for the risk of malnutrition among the research subjects. A total of 256 subjects were judged to have good nutritional status, accounting for 68.63%, while 117 subjects (combining those at risk of malnutrition and those with definite malnutrition) were judged to have malnutrition, accounting for 31.37% (Table 1).

**Table 1:** The nutrition status of research subjects based on MNA questionnaire assessment.

Phase	The score of MNA	Subjects (n)	Proportion (%)	Nutrition status
I	Score ≥ 12	220	58.98	Good nutrition
	Score < 12	153	41.02	Potentially malnutrition
II	Total score ≥ 24	36	9.65	Good nutrition
	17 ≤ total score < 24	96	25.74	Risk of malnutrition
	Total score<17	21	5.63	Definite malnutrition

### 3.3 Univariate analysis

A univariate logistic regression analysis was performed, and the results showed that gender, age, types of elderly care institutions, the presence of geriatric syndromes, frailty, consumptive diseases, peptic ulcers, activity capacity, and neuropsychological problems were statistically significant factors related to malnutrition in the elderly individual (Table 2).

**Table 2:** Univariate logistic regression analysis of malnutrition among elderly individuals.

Item	Number of subjects	Malnutrition rate (%)	OR (95%CI)	*χ* ^2^	*P-*value
Gender				9.53	0.00*
Male	197	24.37	1.00		
Female	176	39.20	2.00(1.28 ~ 3.12)		
Age				11.76	0.01*
60 ~ 69	34	14.71	1.00		
70 ~ 79	91	25.27	1.96(0.68 ~ 5.66)		
80 ~ 89	188	32.98	2.85(1.05 ~ 7.73)		
≧90	60	45.00	4.75(1.62 ~ 13.93)		
Elderly care institution attributes				17.62	0.00*
Publicly constructed and publicly owned	111	32.43	1.00		
Publicly constructed and privately owned	154	26.62	0.76(0.44 ~ 1.29)		
Privately run and publicly owned	55	20.00	0.52(0.24 ~ 1.13)		
Privately run and privately owned	53	54.72	2.52(1.29 ~ 4.92)		
Metabolic syndrome				2.02	0.16
Yes	52	23.07	1.00		
No	321	32.71	0.62(0.31 ~ 1.23)		
Cardiovascular and cerebrovascular diseases				0.63	0.43
Yes	212	33.02	1.00		
No	161	29.19	0.84(0.54 ~ 1.30)		
Mental and psychological diseases				2.56	0.11
Yes	21	47.62	1.00		
No	352	30.40	0.48(0.20 ~ 1.17)		
Liver and kidney diseases				0.03	0.87
Yes	15	33.33	1.00		
No	358	31.28	0.91(0.30 ~ 2.73)		
Peptic ulcer				3.49	0.04*
Yes	35	45.71	1.00		
No	338	29.88	0.51(0.25 ~ 0.98)		
Skeletal system diseases				1.17	0.28
Yes	47	38.29	1.00		
No	326	30.37	0.70(0.37 ~ 1.32)		
Respiratory diseases				2.19	0.13
Yes	27	44.44	1.00		
No	346	30.35	0.55(0.25 ~ 1.20)		
Geriatric syndrome				5.72	0.02*
Yes	39	48.72	1.00		
No	334	29.34	0.44(0.22 ~ 0.86)		
Consumptive diseases				3.57	0.04*
Yes	10	60.00	1.00		
No	363	30.58	0.29(0.08 ~ 0.86)		
Frailty				11.62	0.00*
Yes	19	68.42	1.00		
No	354	29.38	0.19(0.07 ~ 0.52)		
Activity capacity				97.39	0.00*
Bedridden	33	93.94	1.00		
Unable to go out independently	78	51.28	0.07(0.02 ~ 0.30)		
Go out independently	262	17.56	0.14(0.00 ~ 0.06)		
Neuropsychological problems				48.84	0.00*
Severe cognitive decline or depression	7	85.71	1.00		
Mild cognitive decline	52	69.23	0.38(0.04 ~ 3.38)		
Normal status	314	23.89	0.05(0.01 ~ 0.44)		

*Indicates variables with statistical significance.

### 3.4 Multivariate analysis

Variables that showed statistical significance in the univariate regression analysis were included in the multivariate logistic regression model. The results indicated that age, types of elderly care institutions, activity capacity, and the presence of peptic ulcers were related to malnutrition in the elderly (Table 3).

**Table 3:** Multivariate logistic regression analysis of malnutrition among elderly individuals.

Item	OR (95%CI)	*p*-value
Age		0.03*
60–69	1.00	
70–79	4.28(1.02 ~ 17.94)	
80–89	5.48(1.36 ~ 22.07)	
≧90	9.14(2.03 ~ 41.14)	
Elderly care institution attributes		0.01*
Publicly constructed and owned	1.00	
Publicly constructed and privately owned	0.75(0.39 ~ 1.46)	
Privately run and publicly owned	0.19(0.07 ~ 0.52)	
Privately run and owned	0.72(0.27 ~ 1.91)	
Activity capacity		0.00*
Bedridden	1.00	
Unable to go out independently	0.08(0.02 ~ 0.38)	
Go out independently	0.01(0.00 ~ 0.07)	
Peptic ulcer		0.04*
Yes	1.00	
No	0.39(0.16 ~ 0.98)	

*Indicates variables with statistical significance.

## 4 Discussion

The proportion of elderly individuals with malnutrition in the selected elderly care institutions for this study accounted for 31.37% of the total. Various studies indicated differing rates of malnutrition among the elderly population (15–18). The malnutrition rate among elderly individuals found in this study is higher than that of some other regions of China (19, 20). This difference is likely closely related to the economic status and social development of various regions. The dietary intake of elderly individuals is somewhat limited, and their malnutrition is often manifested by a lack of protein, energy, and other nutrients. In economically developed cities, elderly individuals have more opportunities to consume sufficient amounts of nutrients. Elderly care institutions should prioritize regular monitoring, analysis, and evaluation of the nutritional status of the elderly individuals in their care, and make corresponding dietary improvements based on identified issues.

This study found that as age increases, the risk of malnutrition among elderly individuals also increases. This finding is consistent with those of several domestic studies (19, 20), but there are also research results indicating that the risk of malnutrition among elderly individuals in care institutions is not related to age (16). As people age, the physiological functions of the human body gradually decline, and limitations in chewing and digestive functions hinder the elderly from consuming sufficient food. Additionally, some elderly individuals suffered from multiple chronic diseases, and needed to take several medications. Multiple medications can affect the digestion and absorption of food, leading to impaired absorption, utilization, or excretion of nutrients, which increases the risk of malnutrition (21).

The results of this study indicate that different types of elderly care institutions significantly impact malnutrition among the elderly. Findings from multiple factor analyses show that the risk of malnutrition among elderly individuals in privately run and publicly owned care institutions is 0.19 times that of those in publicly constructed and owned care institutions. There are survey results from China indicating that the risk of malnutrition among elderly individuals in care institutions located in urban areas is lower than that in rural areas (19). The impact of elderly care institutions on malnutrition among the elderly is partly related to the economic circumstances of the elderly individuals they serve. Some elderly individuals face limited financial resources and a lack awareness about nutrition and health, which often results in their food demand remaining at a basic level, primarily focused on having meals to avoid starvation. On the other hand, this issue is related to the management abilities of care institution managers. Some care institutions lack professional management training and guidance on elderly nutrition and health, and there is a general lack of awareness regarding professional elderly care.

There is a correlation between the physical activity of elderly individuals and malnutrition. Those who are able to engage in outdoor activities have a relatively lower risk of malnutrition. Lack of physical activity is an important contributing factor to malnutrition (22). The research shows that the risk of malnutrition is most strongly associated with whether elderly individuals are long-term bedridden and whether they are able to eat on their own. Limited physical activity often leads to social isolation, which can result in a decreased appetite and an increased likelihood of malnutrition (23). For example, it is important to conduct regular nutritional screenings for elderly individuals who rely on physical function and to provide sufficient dietary and/or tableware assistance for those with limited physical abilities, such as grasping specially designed tableware that makes eating easier. For elderly individuals with dysphagia, a thorough swallowing assessment should be conducted to tailor intervention measures, such as changing food texture or liquid consistency to match individual swallowing ability. These supportive measures may improve food intake and nutritional status. Additionally, providing a physical rehabilitation program may also have beneficial effects on nutritional status (24, 25).

There is a correlation between peptic ulcer with malnutrition among elderly individuals in our study. Previous studies have demonstrated that peptic ulcer is associated with malnutrition (26–28). The impact of peptic ulcer is not directly causing malnutrition, but rather through discomfort symptoms such as eating pain, acid reflux, and bloating caused by the disease itself, reducing patients’ acceptance and willingness to consume some kinds of foods. Long term exposure may lead to insufficient intake of high-quality proteins, vitamins, and other nutrients.

This study has certain limitations, as there are many types of elderly care institutions, including nursing homes, welfare homes, and rehabilitation hospitals, each with varying admission standards and service capabilities for elderly individuals. This study selected only one elderly care institution from each county in Jiaxing City, and the relatively small number of elderly individuals surveyed may result in selection bias in sample representation. The survey was conducted using a questionnaire survey, and biological samples were not collected for nutrient determination. Information on the dietary intake and social support of elderly individuals in caring institutions has not been thoroughly investigated.

## Data Availability

The original contributions presented in the study are included in the article/supplementary material, further inquiries can be directed to the corresponding authors.
